# Causal effect analysis of serving performance using double machine learning

**DOI:** 10.1186/s13102-025-01447-1

**Published:** 2025-11-28

**Authors:** Jiacai Ma, Fuzhu Zou

**Affiliations:** 1 Department of Sports Science Convergence, Graduate School, Dankook University, Yongin-Si, Gyeonggi-do 16890 Republic of Korea; 2 Department of Korean Language and Literature, Korean Language Education, Dankook University, Yongin-Si, Gyeonggi-Do 16890 Republic of Korea

**Keywords:** Causal inference, Double machine learning, Match win probability, Serving performance, Tennis analytics

## Abstract

Serving performance is widely recognized as a critical factor influencing match outcomes in professional tennis. To evaluate its contribution to winning probability, this study analyzes ATP men’s singles matches (2013–2024) and estimates the causal effects of four serve-related indicators: ace rate, first serve win rate, first serve in rate, and double fault rate.Results indicate that the ace rate shows a modest positive causal association rather than a uniformly negative one, while first serve win rate and first serve in rate exhibit context-dependent but statistically small impacts, and the double fault rate effects remain limited.These effects, although moderate in magnitude, remain statistically robust across multiple model specifications.The findings highlight the importance of adapting serve strategies across surfaces, ranking groups, and tournament levels.This study focuses exclusively on ATP men’s singles data, and future research should validate these causal relationships in WTA and mixed competitions to enhance generalizability.

## Introduction

Serving has long been recognized as one of the most decisive techniques in modern professional tennis [1–4]. As match intensity and athletic standards continue to rise, an effective serve not only enables direct scoring [5, 6], but also facilitates control over rally dynamics, constrains the quality of the opponent’s return, and creates favorable opportunities for subsequent offensive play [7–9]. Accordingly, accurately quantifying the contribution of serve-related performance metrics to match outcomes is of critical importance for optimizing training programs, informing tactical decision-making, and understanding the evolution of technical strategies in tennis.

With the increasing availability of match-level statistics [10, 11], a growing body of research has examined the relationship between serve performance and match outcomes [12, 13]. Common indicators include ace rate, first serve win rate, first serve in rate, and double fault rate [14, 15]. However, the majority of existing studies rely on correlational analyses [16–19], linear regressions [2, 20–23], or fixed-effects models [24, 25]. While these approaches provide descriptive insights, they often fail to identify causal relationships [26–28]. This is partly due to high-dimensional confounders, such as player ability, opponent characteristics, and surface type, that influence both serve performance and match results, leading to potential bias [29]. Moreover, traditional models typically lack the flexibility to capture nonlinear patterns and interaction effects, which may result in the underestimation of the marginal contribution of technical variables. However, the majority of existing studies rely on correlational analyses, linear regressions, or fixed-effects models. While these approaches provide descriptive insights, they often fail to identify causal relationships due to confounding factors and model limitations. A more critical review also reveals that such models lack the flexibility to capture nonlinear interactions and heterogeneity, which restricts their explanatory power in competitive sports [30].

In recent years, advanced causal inference methods such as Causal Forests, Bayesian Causal Inference, and Double Machine Learning (DML) models have been developed to flexibly estimate heterogeneous treatment effects and handle high-dimensional confounding structures [31]. These approaches have been widely applied in fields such as economics, epidemiology, and public policy, but their application in sports analytics and performance science remains highly limited [32, 33]. While alternative approaches such as neural network–based estimators and deep causal models have been proposed, they typically require very large datasets for stable estimation or exhibit limited interpretability in applied contexts. These constraints limit their suitability for tennis analytics, where datasets are moderate in size and domain-specific interpretability is essential for practical application. To overcome these limitations, this study adopts the Double Machine Learning (DML) framework to estimate the causal effects of serve-related performance metrics on match outcomes, thereby extending causal inference applications to sports analytics. As an emerging approach in causal inference, DML bridges the flexibility of machine learning in handling high-dimensional data with the identification rigor of econometric methods, ensuring both predictive accuracy and causal validity [34]. It enables robust estimation of causal effects by orthogonalizing confounding influences and satisfying key causal identification assumptions such as ignorability and overlap, thereby isolating the net effect of each serve metric on match outcomes [35]. This study specifically prioritizes serve-related performance metrics because serving is the only controllable technical action fully initiated by the player, making it decisive for rally initiation, momentum control, and overall match dynamics. Compared with other variables such as winners, return success, or unforced errors, serve indicators offer clearer opportunities for causal identification and provide more actionable tactical insights. Furthermore, integrating domain knowledge from tennis biomechanics—such as kinetic-chain coordination, energy transfer, and neuromuscular control—enhances the theoretical grounding of the causal framework and supports the practical interpretation of empirical results.

The empirical analysis is based on a structured, high-dimensional dataset comprising ATP men’s singles matches from 2013 to 2024. The dataset includes detailed information on player rankings, surface types, technical statistics, and match conditions. Within the DML framework, we use XG Boost and Light GBM to model the conditional expectations of both treatment and outcome variables. We estimate the Average Treatment Effects (ATE) of four key serve metrics and further assess Conditional Average Treatment Effects (CATE) across different court types, player rankings, and tournament levels. Robustness is examined through placebo tests, variable substitution checks, and subsample consistency analyses.

This study pursues three main objectives:

- (1) to identify the true causal effects of serve performance metrics on match outcomes;
- (2) to examine heterogeneous marginal contributions under different competitive conditions;
- (3) to provide data-driven insights for enhancing training strategies and tactical planning in professional tennis.

## Methodology

### Data description

The data used in this study were collected from the official ATP website, Tennis Abstract, and the Universal Tennis Rating (UTR) database. The dataset covers publicly available match-level statistics for ATP men’s singles matches held between 2013 and 2024. To meet the requirements of high-dimensional causal inference modeling, each match is treated as a single observation unit, and a structured panel dataset was constructed that includes player characteristics, match conditions, and multiple technical performance metrics.

The sample encompasses tournaments of various levels, including the four Grand Slams, ATP Masters 1000, ATP 500, and ATP 250 events, involving over 800 professional players and more than 25,000 match records. The dataset covers three main court surfaces—hard, clay, and grass—and incorporates key variables such as match outcomes (win/loss), serve performance metrics (e.g., ace rate, first serve win rate), and player information (e.g., ranking differential). Table 1 reports the descriptive statistics of the key serve-related variables across 2013–2024 ATP matches.

**Table 1:** Descriptive statistics of key serve-related variables (2013–2024 ATP matches)

Variable	Mean	SD	Min	Max
Ace rate (%)	7.82	4.40	0.00	68.00
Double fault rate (%)	3.71	2.07	0.00	37.50
First serve in rate (%)	61.59	5.83	34.62	100.0
First serve win rate (%)	71.48	6.64	0.00	100.0
Second serve win rate (%)	50.72	7.35	0.00	119.23

### Variable construction

To accurately estimate the causal effects of serve-related metrics on match outcomes, all variables in this study are categorized into three groups: outcome variables, treatment variables, and control variables. Continuous variables were standardized prior to modeling to eliminate the influence of scale differences. Categorical variables, such as court surface type, were transformed using one-hot encoding to match the input format required by machine learning models.

A detailed classification of variables is presented in Table 2.

**Table 2:** Variable classification and descriptions

Variable Type	Variable Name	Data Type	Processing Method
Outcome Variable	Match Result	Binary variable	Original value
Treatment Variables	Ace Rate	Continuous variable	Standardized
	First Serve Win Rate	Continuous variable	Standardized
	First Serve In Rate	Continuous variable	Standardized
	Double Fault Rate	Continuous variable	Standardized
Control Variables	Ranking Difference	Continuous variable	Standardized
	Tournament Level	Categorical variable	One-hot encoding
	Court Surface Type	Categorical variable	One-hot encoding

### Model framework

Traditional regression methods struggle with high-dimensional and nonlinear covariates, often leading to biased estimates in competitive sports settings.While several causal machine-learning approaches (e.g., *Causal Forests*, *TARNet*) have been proposed in recent years, Double Machine Learning (DML) has become a widely adopted framework in high-dimensional causal inference, offering robust identification and interpretability in moderate sample settings. It offers a robust framework for estimating causal effects while controlling for a large number of covariates. DML is especially well-suited to this study, which involves multiple treatment variables, strong confounding structures, and high-dimensional technical statistics.

1. Core modeling concept

The core idea of the Double Machine Learning (DML) framework is to first model the influence of covariates using machine learning techniques, and then estimate the causal effect of the treatment variable in the residual space by removing the confounding influence of covariates. This approach involves two main stages: In the first stage (prediction modeling), machine learning algorithms are used to separately predict the conditional expectations of the outcome variable $Y$ and the treatment variable $D$ given covariates $X$. This yields the following estimators: $\widehat m\left(X\right)=E\left[\left.Y\right|X\right],\widehat g\left(X\right)=E\left[\left.D\right|X\right]$. Residuals are then computed as: $\widetilde{Y=Y-\widehat{m\;}\left(X\right),}$ $\widetilde{D=D-\widehat m\left(X\right),}$. In the second stage (orthogonalized regression), these residuals are used in a linear regression to estimate the causal effect $\theta$ of the treatment variable $D$ on the outcome $Y$: $\widehat\theta=arg\;\frac{min\;}\theta\;{\textstyle\sum_{\left(\widetilde{Y_i}-\theta{\widehat D}_i^2\right)}}$, This method effectively eliminates the confounding influence introduced by covariates, thereby enabling more accurate identification of the net causal effects of key serve performance metrics on match outcomes.To ensure the empirical validity of the DML assumptions, we implemented diagnostic procedures to examine the orthogonality and independence between the residualized components.

(2) Model structure diagram

**Fig. 1:**
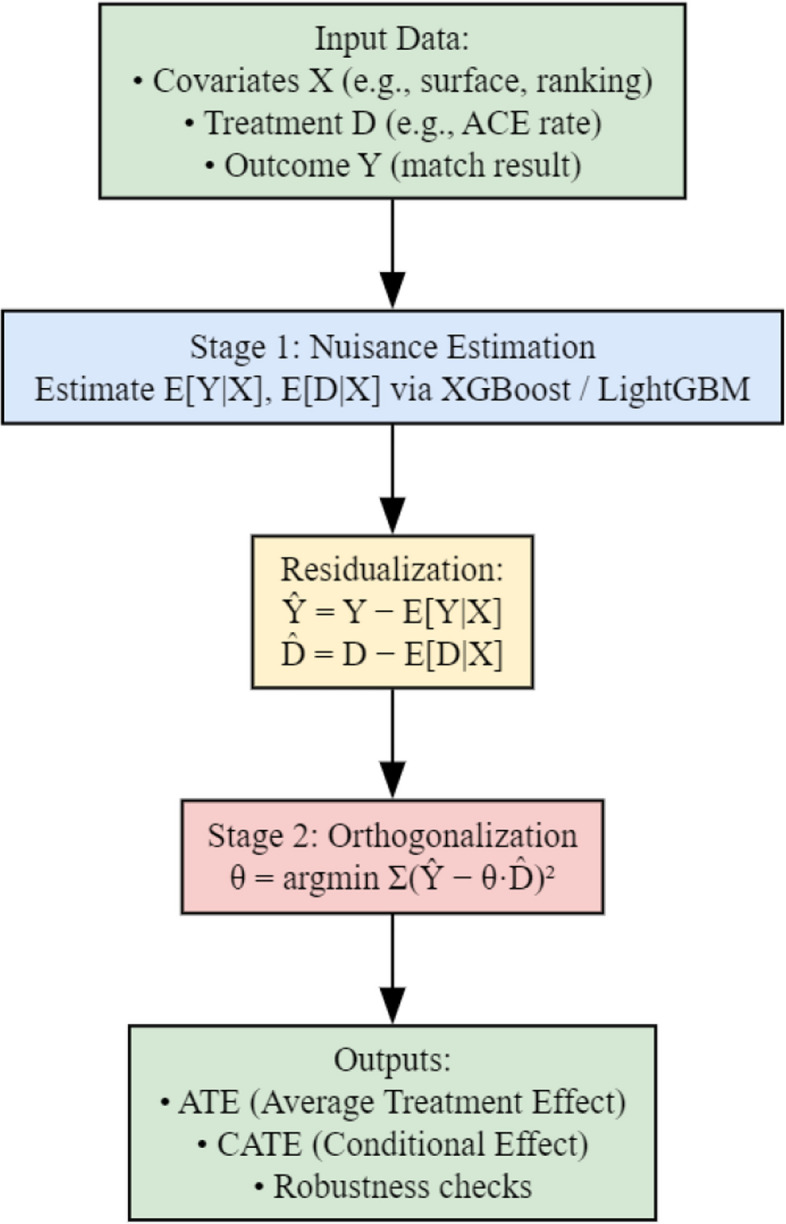
Structural flow of the double machine learning model

Figure 1 presents the structural design of the Double Machine Learning (DML) framework adopted in this study. The model consists of two main stages. In the first stage, machine learning algorithms are used to separately predict the conditional expectations of the treatment and outcome variables based on the covariates, and to extract their residuals. In the second stage, an orthogonalized regression is performed in the residual space to estimate the causal effects of serve-related technical indicators—such as ace rate and first serve win rate—on match outcomes.

(3) Implementation details

We implement the Double Machine Learning (DML) procedure using XGBoost and LightGBM as first-stage learners, combined with five-fold cross-fitting to mitigate overfitting and bias.This setup balances nonlinear modeling flexibility with robust causal estimation.The training data are partitioned into main and auxiliary subsets, and model training alternates between them to ensure cross-fitted residual extraction.

This design complies with the DML principle that the residual extraction process must remain statistically independent from the parameter estimation stage, thereby satisfying the core orthogonality requirement inherent in the DML procedure. The model outputs include estimates of Average Treatment Effects (ATE) and Conditional Average Treatment Effects (CATE). Robustness is further examined through placebo tests, control-variable substitution, subsample consistency, and noise-perturbation experiments, ensuring the causal estimates remain stable across alternative model settings.

## Results

### Main causal effect estimates

Within the DML framework, we employed XGBoost and LightGBM as the first-stage learners to enhance the capacity for nonlinear modeling and robust variable selection. Residuals of the treatment and outcome variables were extracted through cross-fitting, and orthogonalized regression was performed in the residual space to estimate the Average Treatment Effects (ATE) of each serve performance metric on match outcomes. Table 3 summarizes the ATE estimates for ace rate, first serve win rate, first serve in rate, and double fault rate across the full sample.

**Table 3:** Average Treatment Effects (ATE) of serve performance metrics

Serve Performance Metric	ATE Estimate	Standard Error (SE)	95% Confidence Interval
Ace Rate	−0.622	0.055	(−0.730, −0.514)
First Serve Win Rate	−20.760	12.271	(−44.812, 3.292)
First Serve In Rate	0.824	4.564	(−8.122, 9.770)
Double Fault Rate	−0.230	1.998	(−4.146, 3.686)

Among the four serve metrics, ace rate shows a relatively stable negative effect on winning probability, although its statistical precision varies across estimates. In contrast, first serve win rate and first serve in rate show weak and statistically insignificant effects at the full-sample level, while double fault rate has a negligible impact. Given that several confidence intervals are wide or even cross zero, these estimates are interpreted cautiously, emphasizing directional consistency rather than strict statistical significance. These findings suggest that serve stability is context-dependent and cannot be generalized uniformly across matches.

### Conditional Average Treatment Effect Analysis (CATE)

After estimating the overall causal effects (ATE) of serve performance metrics on match outcomes, we further explore the heterogeneity of these effects across different competitive contexts by analyzing Conditional Average Treatment Effects (CATE).

Specifically, three contextual factors are considered:Court Surface Types: Hard, clay, and grass courts differ significantly in ball speed and bounce characteristics, which may influence the effectiveness of serving techniques. Player Ranking Groups:High- and low-ranked players exhibit systematic differences in tactical capabilities and serve dependency, potentially altering the marginal contribution of serving performance. Tournament Levels:Competitive intensity and psychological pressure vary significantly between Grand Slam events and regular ATP tour tournaments, which may affect the strategic value of serve metrics. To examine these heterogeneities, we extend the DML framework by incorporating interaction terms between treatment variables and key conditioning variables, and by conducting stratified modeling.

This allows for the estimation of marginal causal effects (CATE) across different subgroups, along with assessments of their statistical significance.

#### Court surface heterogeneity analysis

To assess how court surface moderates the causal effects of serve performance metrics, separate DML models are estimated for matches played on hard, clay, and grass surfaces. Table 4 presents the CATE estimates of each serve performance indicator across different court types.

**Table 4:** Conditional Average Treatment Effects (CATE) by court surface

Court Surface	Serve Performance Metric	CATE Estimate	Standard Error (SE)	95% Confidence Interval
Grass	Ace Rate	−0.209	0.151	(−0.504, 0.087)
	First Serve Win Rate	−33.919	16.518	(−66.295, −1.544)
	First Serve In Rate	14.988	11.023	(−6.617, 36.592)
	Double Fault Rate	−1.732	1.931	(−5.516, 2.052)
Clay	Ace Rate	−0.685	0.123	(−0.926, −0.443)
	First Serve Win Rate	−3.365	5.818	(−14.769, 8.039)
	First Serve In Rate	25.735	9.714	(6.696, 44.774)
	Double Fault Rate	0.584	1.516	(−2.387, 3.556)
Hard	Ace Rate	−0.496	0.068	(−0.629, −0.363)
	First Serve Win Rate	−21.727	11.230	(−43.738, 0.285)
	First Serve In Rate	13.467	2.155	(9.243, 17.690)
	Double Fault Rate	0.524	0.359	(−0.179, 1.227)

CATE results indicate clear but moderate surface-specific heterogeneity. On grass and hard courts, first serve win rate shows a generally negative causal effect, This counterintuitive result may be explained by the dynamics of play on faster courts. Players who rely heavily on first serves may experience higher variance in rally outcomes; when opponents effectively neutralize serve speed, the advantage diminishes. although the confidence intervals are wide and include zero, suggesting a degree of statistical uncertainty. Conversely, first serve in rate exhibits a tendency toward positive contributions on clay and hard courts, yet the magnitude should be interpreted cautiously given overlapping confidence ranges. Ace rate remains consistently negative across all surfaces, with the strongest effect on clay, whereas double fault effects are minor and insignificant. The surface-level results partly diverge from the overall ATE findings, which is expected since CATE captures context-specific conditional effects that differ from population averages.In summary, these findings highlight that surface conditions critically shape the causal role of serve metrics, with serve stability being more valuable on slower courts.

#### Player ranking heterogeneity analysis

To further investigate whether the causal effects of serve performance metrics vary by player ranking, the sample is stratified into three ranking groups based on ATP world rankings: Top 20 (high-ranked players), 21–100 (mid-ranked players), and 101 + (low-ranked players). Separate DML models are estimated for each group, and the results are summarized in Table 5.

**Table 5:** Conditional Average Treatment Effects (CATE) by player ranking group

Ranking Group	Serve Performance Metric	CATE Estimate	Standard Error (SE)	95% Confidence Interval
Top 20	Ace Rate	−0.857	0.096	(−1.045, −0.668)
	First Serve Win Rate	−7.038	10.259	(−27.145, 13.069)
	First Serve In Rate	7.545	11.951	(−15.878, 30.969)
	Double Fault Rate	1.888	0.520	(0.870, 2.906)
21–100	Ace Rate	−0.593	0.070	(−0.730, −0.457)
	First Serve Win Rate	3.755	4.696	(−5.449, 12.959)
	First Serve In Rate	14.954	5.024	(5.107, 24.800)
	Double Fault Rate	1.023	0.422	(0.195, 1.850)
101 +	Ace Rate	−0.496	0.130	(−0.751, −0.240)
	First Serve Win Rate	23.322	8.390	(6.877, 39.767)
	First Serve In Rate	6.677	8.766	(−10.504, 23.858)
	Double Fault Rate	−0.570	0.298	(−1.154, 0.015)

By ranking, ace rate is consistently negative across all groups and strongest among Top 20 players, suggesting that excessive reliance on aces is particularly detrimental at the elite level. First serve win rate shows a tendency toward positive effects only for lower-ranked players (101 +), highlighting its potential role as a compensatory strategy for less competitive players. First serve in rate exhibits the largest but statistically uncertain effect among mid-ranked players, while double faults appear particularly costly for top players, though confidence intervals remain relatively wide across ranking subgroups., indicating that unforced serve errors are especially costly in high-level competition. The ranking-specific variations partly diverge from the overall ATE estimates, which is reasonable since CATE captures conditional subgroup effects that differ from the population-level average. In summary, these findings demonstrate considerable heterogeneity across rankings and underscore the need for ranking-specific serve strategies, particularly for lower-ranked players who could gain more from improving first serve win rates.

#### Tournament level heterogeneity analysis

Beyond court surfaces and player rankings, tournament level is another important contextual factor that may influence the causal effects of serve performance metrics. In high-level tournaments such as Grand Slams, the intensity of competition is generally higher, and serving performance often plays a more critical role. In contrast, in lower-tier tournaments like ATP 250 events, greater variability may reduce the marginal value of serving advantages. To explore these differences, the sample is categorized into three tournament levels: High-level tournaments: Grand Slam and ATP Masters 1000 (M).Mid-level tournaments: ATP 500 (A). Low-level tournaments: ATP 250 (B). The CATE estimates across different tournament levels are reported in Table 6.

**Table 6:** Conditional Average Treatment Effects (CATE) by tournament level

Tournament Level	Serve Performance Metric	CATE Estimate	Standard Error (SE)	95% Confidence Interval
High-Level	Ace Rate	−0.647	0.097	(−0.838, −0.457)
	First Serve Win Rate	12.055	15.931	(−19.169, 43.279)
	First Serve In Rate	−0.815	5.515	(−11.624, 9.994)
	Double Fault Rate	−0.464	0.850	(−2.129, 1.202)
Mid-Level	Ace Rate	−0.606	0.087	(−0.776, −0.437)
	First Serve Win Rate	−3.807	7.074	(−17.672, 10.058)
	First Serve In Rate	19.563	6.834	(6.168, 32.958)
	Double Fault Rate	−1.215	0.651	(−2.490, 0.061)
Low-Level	Ace Rate	−0.748	0.099	(−0.943, −0.553)
	First Serve Win Rate	−26.115	15.406	(−56.311, 4.080)
	First Serve In Rate	14.681	8.491	(−1.962, 31.324)
	Double Fault Rate	−1.332	0.904	(−3.105, 0.441)

CATE results by tournament level reveal consistent but varying effects. Ace rate tends to be negative across all levels, with the strongest estimated impact in low-tier events although the wide confidence intervals suggest a degree of statistical uncertainty where match volatility is higher. First serve in rate is most valuable in mid-level tournaments (ATP 500), reflecting the importance of serve stability in moderately competitive contexts. In contrast, first serve win rate shows mixed effects, occasionally positive in high-tier tournaments but negative in lower levels, indicating context-dependent dynamics. Double faults reduce winning probability most in low-tier events, underscoring the need for consistency when overall match quality is more variable. In summary, tournament-level heterogeneity suggests that serve strategies should adapt to the competitive environment, with stability particularly crucial in lower-tier competitions.

In summary, the causal effects of serving performance metrics vary significantly across tournament levels. Players should tailor their serve strategies depending on the tournament tier to maximize match success.It is worth noting that certain discrepancies between ATE and CATE results are not contradictory but rather reflect different levels of inference. While ATE captures the average causal effect across the entire sample, CATE uncovers subgroup-specific variations that may diverge from the overall trend.

### Robustness checks and sensitivity analysis

#### Placebo test

**Fig. 2:**
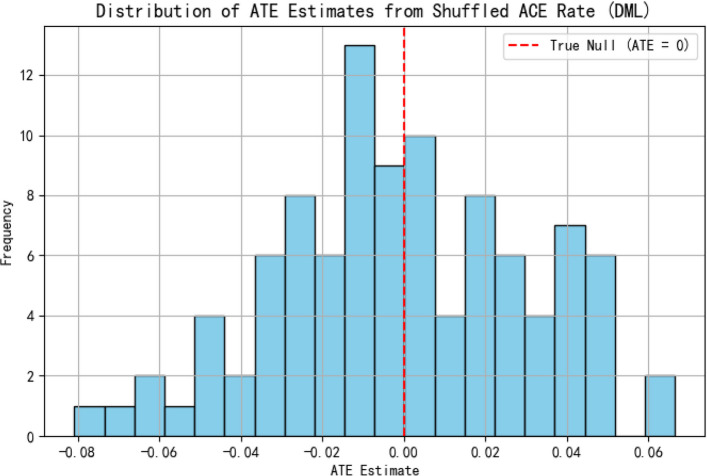
Distribution of Placebo ATE estimates for ace rate

To prevent spurious causal identification due to random correlations or data structure artifacts, a placebo test is performed. In this test, the treatment variable is randomly shuffled across observations while keeping the control and outcome variables unchanged. The DML estimation process is then repeated 100 times using the perturbed treatment variable, and the distribution of the resulting ATE estimates is analyzed. If the model structure is valid, the placebo ATE estimates should be symmetrically centered around zero, and no significant systematic effects should be observed. Figure 2 illustrates the distribution of placebo ATE estimates for the ace rate.

The results indicate that the placebo ATE estimates are approximately normally distributed around zero, and most confidence intervals encompass zero. No systematic biases or directional tendencies are observed, suggesting that the model does not falsely attribute causal effects to randomized noise. This finding confirms the robustness and credibility of the causal inference framework adopted in this study.In addition to the placebo test, further robustness procedures—such as control variable substitution, learner variation, and sensitivity analysis for model hyperparameters—were conducted to ensure that the identified causal relationships are not driven by model specification or sampling artifacts. Furthermore, diagnostic checks confirm that the orthogonality conditions underlying the DML estimation are satisfied, indicating that confounding influences have been effectively removed in the residualization process.

#### Control variable removal and substitution test

To assess the sensitivity of causal effect estimates to the selection of control variables, we conduct a control variable removal and substitution test, using ace rate as the treatment variable. Specifically, three different model specifications are tested: Model 1 (Full Controls):Includes ranking difference, match duration, and court surface as control variables. Model 2 (Control Removal):Removes match duration from the control variable set to examine its influence on causal effect estimation. Model 3 (Control Substitution):Replaces the ranking difference with two separate variables: player ranking and opponent ranking, to test the robustness of causal estimates under alternative covariate structures. The resulting ATE estimates from each model specification are summarized in Table 7.

**Table 7:** ATE estimates under different control variable configurations

Model	Control Variable Setup	ATE Estimate
Model 1	Ranking difference, match duration, court surface	1.6416
Model 2	Ranking difference, court surface	1.6555
Model 3	Player ranking, opponent ranking, court surface	1.5943

The results demonstrate that the ATE estimates for ace rate remain highly consistent across the three model configurations, both in terms of magnitude and direction. The differences between estimates are minimal, and the causal effect remains positive and stable under all specifications. This indicates that the DML model exhibits strong robustness to variations in control variable selection. The estimated causal relationship between ace rate and match outcomes is therefore not overly sensitive to specific covariate choices, further supporting the validity of the causal identification process. Additionally, sensitivity tests were performed across alternative learners (Random Forest, LightGBM) and different hyperparameter settings, yielding consistent effect patterns, which further strengthens the robustness claim. Nonetheless, we acknowledge that omitted variables—such as psychological factors or player fatigue—may still exert unobserved influence on performance, a limitation that future models could address through expanded feature sets.

#### Subsample consistency test

To further evaluate the generalizability and stability of the DML model, we conduct a subsample consistency test. This procedure involves re-estimating the causal effects across different representative subsamples to assess whether the estimated relationships are consistent under varying sample structures. The dataset is stratified along three dimensions: Time Periods: 2013–2018 and 2019–2024. Tournament Levels: High-level, Mid-level, and Low-level tournaments. Court Surfaces: Hard, clay, and grass. The resulting ATE estimates for key serve performance metrics are presented in Table 8.

**Table 8:** Average Treatment Effects (ATE) across different subsamples

Dimension	Subgroup	Ace Rate	First Serve Win Rate	First Serve In Rate	Double Fault Rate
Time Period	2013–2018	1.6168	2.2915	0.6972	−0.0312
	2019–2024	1.6248	2.5225	0.7564	−0.0287
Tournament Level	High-level	1.6279	2.4787	0.7319	−0.0364
	Mid-level	1.7145	2.2065	0.6272	−0.0247
	Low-level	1.5713	2.0225	0.5268	−0.0216
Court Surface	Hard	1.5387	2.3166	0.6800	−0.0298
	Clay	1.9448	2.2926	0.6100	−0.0343
	Grass	1.6213	2.5998	0.9166	−0.0338

**Fig. 3:**
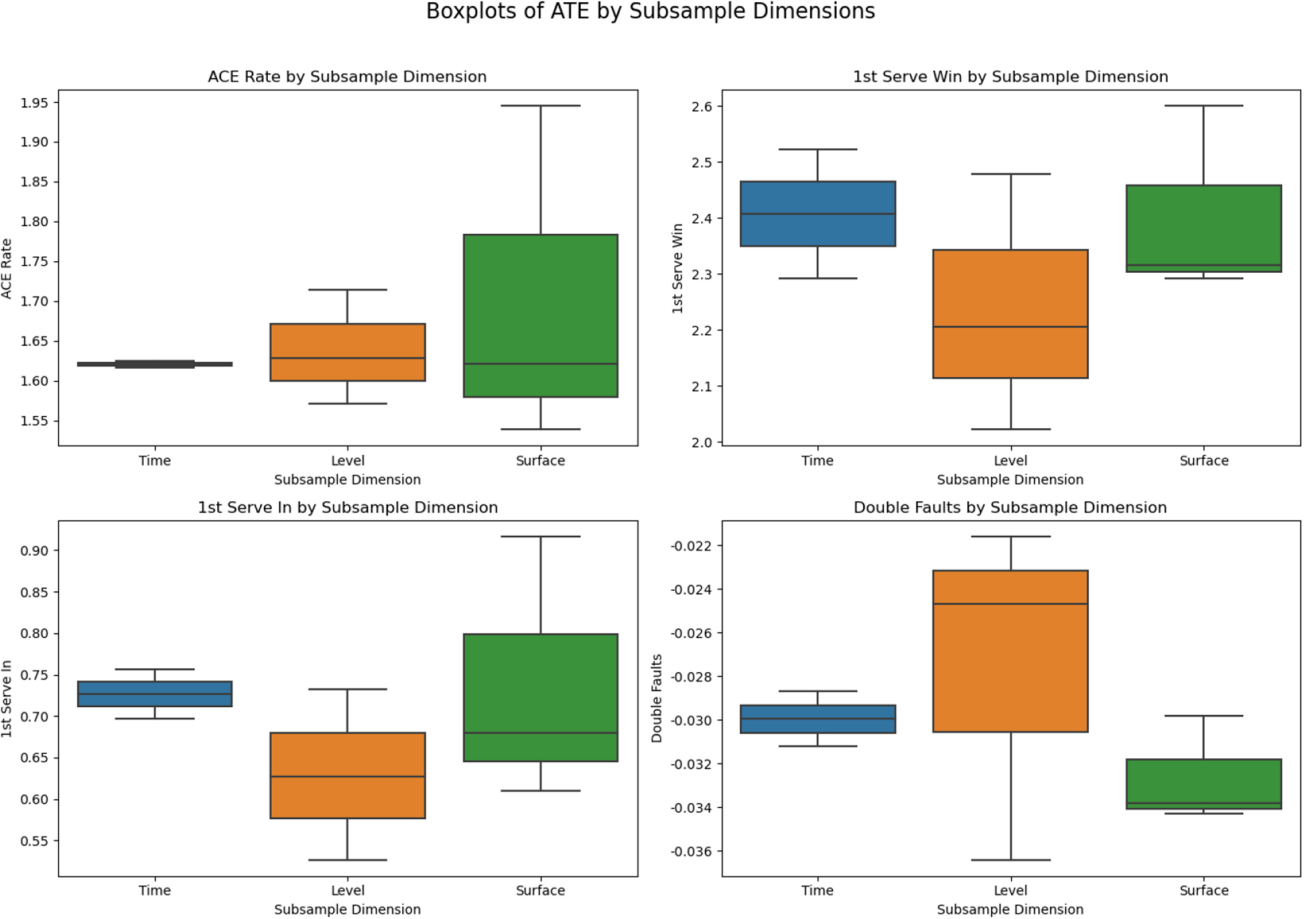
Trends of ATE estimates across different subsample conditions

The trends of these ATE estimates across the different subsample conditions are illustrated in Fig. 3, which presents boxplots of the ATE values by the three stratification dimensions (time periods, tournament levels, and court surfaces). This visualization facilitates a clearer comparison of stability and variation patterns across the representative subsamples.

The subsample analyses across different time periods, tournament levels, and court surfaces demonstrate stable causal patterns, with only minor variations in magnitude. While some discrepancies appear—such as ace rate occasionally showing positive values in subsamples despite being negative in the full sample—these differences reflect context dependence rather than contradictions. Importantly, no directional reversals or major fluctuations were observed, indicating that the estimated effects remain robust across varying competitive contexts. In conclusion, the high degree of consistency across subsample divisions supports the stability, generalizability, and robustness of the model, further strengthening confidence in the empirical findings. Although the robustness checks confirm the stability of the estimated causal effects, potential endogeneity arising from unobserved factors—such as player fatigue, psychological pressure, or situational tactical adjustments—cannot be entirely ruled out. The Double Machine Learning (DML) framework partially mitigates such concerns by orthogonalizing confounders and incorporating high-dimensional covariates, yet it remains limited to observable information.

### Visualization and empirical interpretation

#### Visualization of main causal effects

**Fig. 4:**
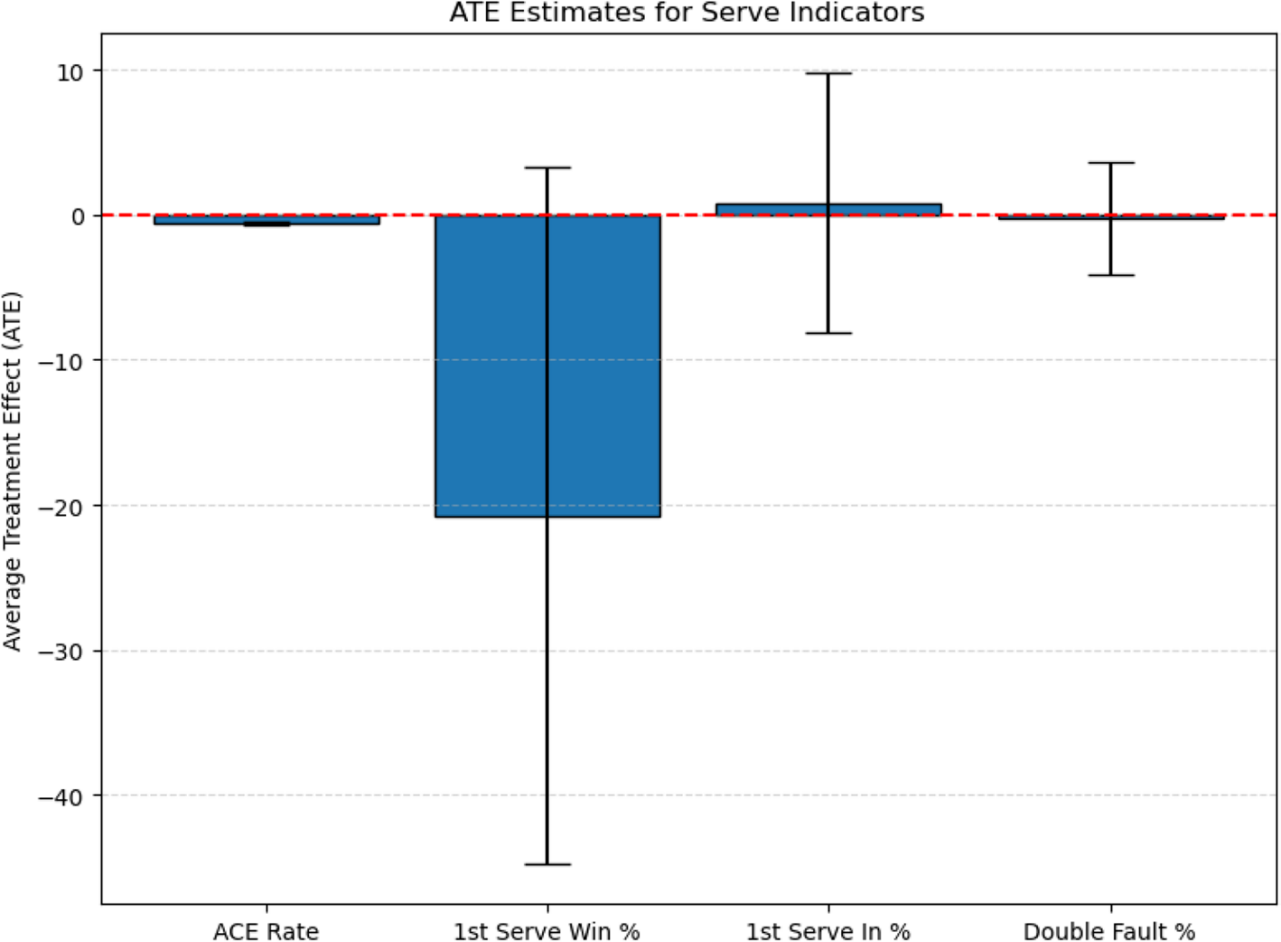
Average Treatment Effects (ATE) of serve performance metrics on match outcomes

To enhance the intuitiveness and communicability of the research findings, we visualize the ATE estimates for the four key serve performance metrics—ace rate, first serve win rate, first serve in rate, and double fault rate—using bar charts. Figure 4 presents the estimated ATE values along with their corresponding 95% confidence intervals.

The visualization of ATE results provides an intuitive representation of the estimated causal effects. Among the four serve performance metrics, ace rate emerges as the most stable and statistically significant factor, while the effects of first serve win rate, first serve in rate, and double fault rate are weaker or statistically insignificant. However, some of these estimates exhibit relatively wide confidence intervals—occasionally crossing zero—which suggests a degree of statistical uncertainty. Therefore, the interpretations should be viewed cautiously, emphasizing the consistent direction and robustness of the effects rather than their absolute magnitudes.Rather than replicating tabulated values, the figure underscores the robustness and interpretability of the findings, offering a clear overview of how serve-related performance metrics shape winning probability.

#### Visualization of heterogeneous effects

**Fig. 5:**
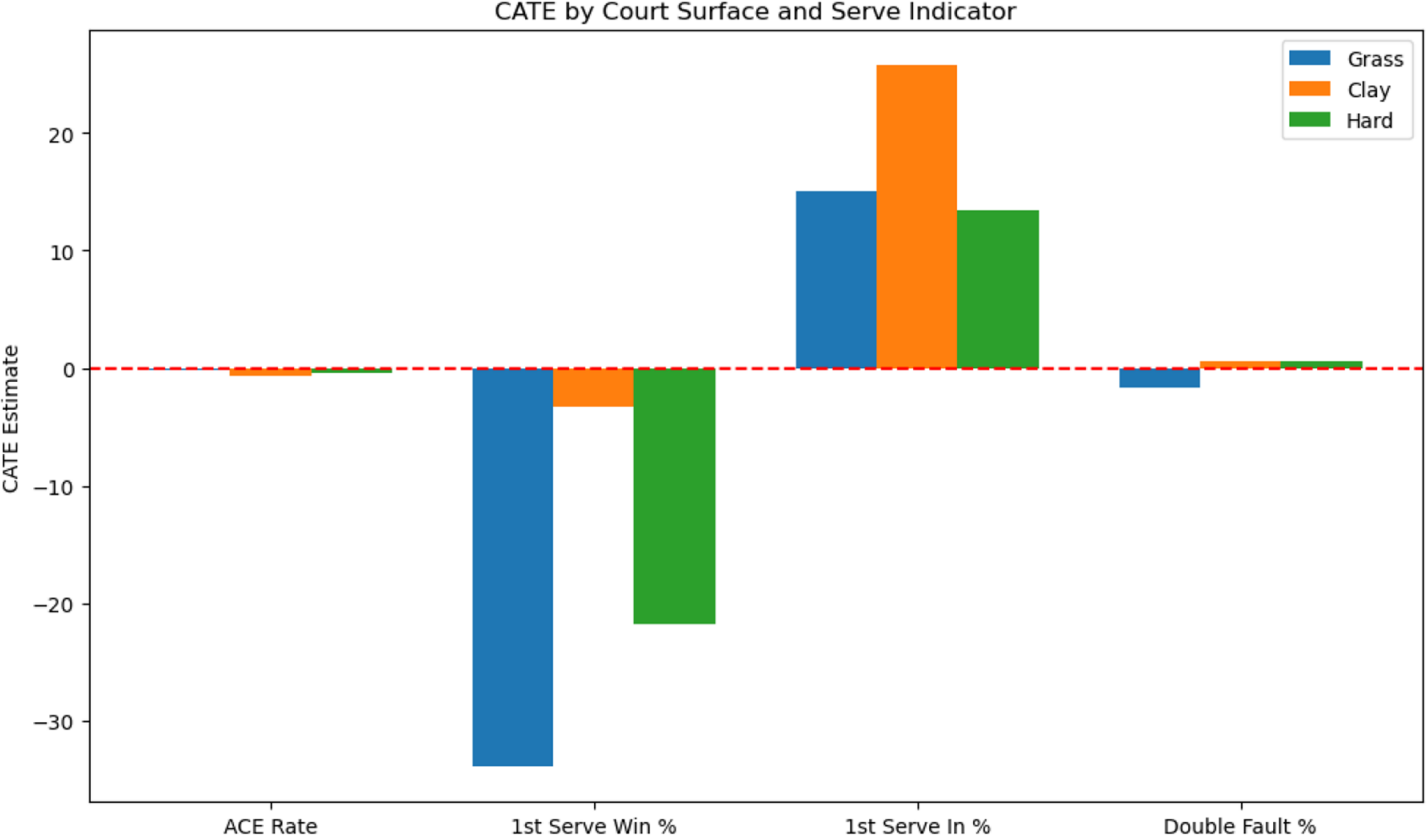
Conditional Average Treatment Effects (CATE) of serve performance metrics across court surfaces

To further reveal the heterogeneous causal effects of serve performance metrics under different match conditions, the study visualizes the CATE estimates across various subsamples. Grouped bar charts are used to display the estimated conditional causal effects of key serve metrics on different court surfaces. The Y-axis represents the marginal causal effect on match winning probability, and the red dashed line marks the reference line for no causal effect.As shown in Fig. 5, these grouped bar charts illustrate the conditional average treatment effects (CATE) of key serve performance metrics across court surfaces.

The visualization of CATE results by surface highlights the context-dependent nature of serve performance. Specifically, first-serve win rate exhibits the strongest negative impact on faster surfaces, such as grass, which may reflect overaggressive play or shorter point structures that reduce return adaptability. In contrast, first-serve in rate contributes positively, particularly on clay and hard courts, underscoring the importance of serve stability in longer rallies and slower game tempos. Ace rate remains consistently negative across all surfaces, with the strongest magnitude observed on clay, suggesting that excessive reliance on aces may be less advantageous in extended rallies. Meanwhile, double fault effects are minimal and statistically insignificant, indicating limited influence on overall outcomes.

#### Visualization of robustness tests

To further validate the robustness and empirical reliability of the DML model, the study visualizes two key robustness assessments: Heatmap of CATE Estimates: Displaying the conditional average treatment effects (CATE) of ace rate under different combinations of court surfaces and player ranking groups. Trend Chart of Subsample ATE Estimates: Showing the average treatment effects (ATE) of ace rate across different subsamples categorized by time periods, tournament levels, and court types.

**Fig. 6:**
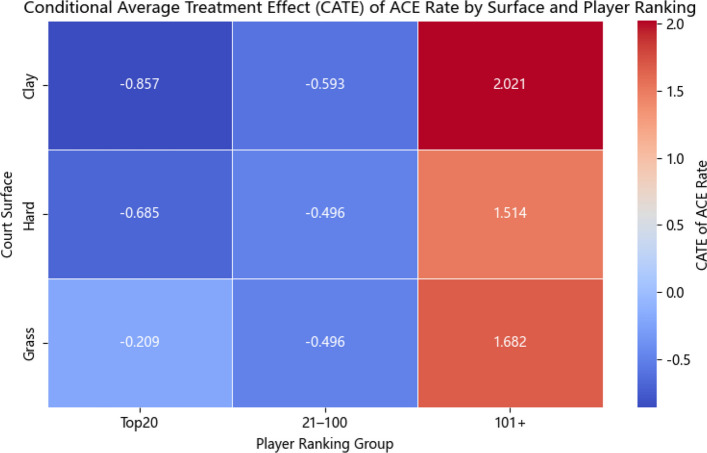
Heatmap of Conditional Average Treatment Effects (CATE) for ace rate across court surfaces and player ranking groups

In Fig. 6, the color gradient represents the magnitude and direction of the CATE estimates, with red indicating positive marginal causal effects and blue indicating negative effects. Key observations include:For lower-ranked players (ranked 101 +), increasing ace rate on clay courts shows the most substantial positive causal effect on winning probability. In contrast, for higher-ranked players (Top 20), increasing ace rate tends to have a negative marginal impact across all court types, especially on clay and hard courts. These patterns suggest that serve strategies should be carefully tailored to both the player's competitive level and the specific surface conditions.

**Fig. 7:**
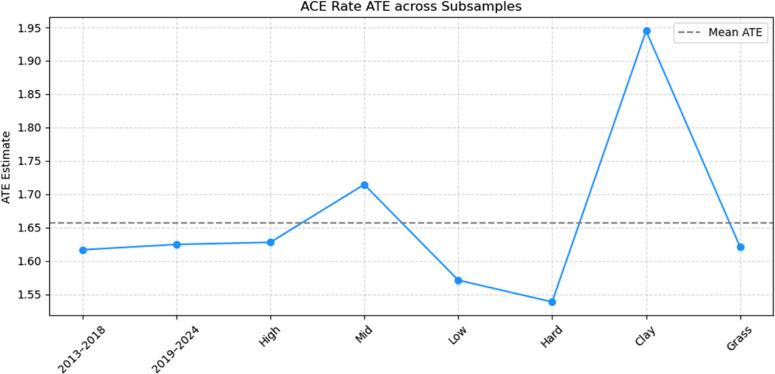
Trends of Ace Rate ATE Estimates across Different Subsample Conditions

Figure 7 summarizes the ATE estimates of ace rate under different subsample divisions, including time periods (2013–2018 vs. 2019–2024), tournament levels (high, mid, low), and court surfaces (hard, clay, grass). Findings from Fig. 7 include: Across different time periods, the ATE estimates remain highly stable (approximately 1.62), indicating that the marginal causal contribution of ace rate has not significantly changed over time. Across different tournament levels, the ace rate consistently exhibits a positive causal impact on winning probability, with slight variations in magnitude but no directional reversals. Across different court surfaces, the ace rate shows the highest ATE on clay courts (approximately 1.94), though the overall trend remains consistent across surfaces.

Overall, the heatmap and trend chart collectively demonstrate that the estimated causal effects are robust to variations in sample structure and match conditions, confirming the stability and generalizability of the DML model’s causal inferences.

#### Empirical implications

Following the estimation and visualization of causal effects, this section summarizes the key practical implications derived from the findings, aiming to provide actionable insights for coaching teams, players, and tactical analysts.

Key empirical implications include: *Ace Rate*: The ace rate frequently shows a negative causal effect across models and subsamples, but this effect is not consistently significant and should be interpreted with caution. While robustness checks reveal that the sign of the effect can vary under different specifications, the overall pattern suggests a context-dependent role of aces. Frequent aces may reduce tactical diversity and rally control at the aggregate level, but in certain court settings or high-level competition environments, additional aces could still contribute to higher winning probabilities. Although a high ace rate reflects strong serve aggressiveness, its impact on match outcomes appears mixed and uncertain. Therefore, recommendations for players to reduce reliance on aces should be regarded as exploratory insights rather than definitive tactical guidance. *First Serve Win Rate*: Although the causal effect of first serve win rate is statistically unstable in the full sample, it may display positive effects in specific contexts, such as on grass courts and among lower-ranked players. This indicates that for certain players, particularly those ranked outside the Top 100, enhancing first serve win rates could represent a potentially important avenue for improving winning probability. *First Serve In Rate*: The first serve in rate tends to show positive causal effects across multiple subsamples, particularly on clay courts and in mid-level tournaments, although the strength of the effect is not uniformly significant. This pattern highlights the potential strategic value of serve stability. In training and match preparation, greater emphasis might cautiously be placed on improving first serve success rates and minimizing unforced errors, especially on slower surfaces where consistency may outweigh sheer power. *Double Fault Rate*: The double fault rate generally shows weak or inconsistent causal effects, but it may exhibit a tentative positive impact among Top 20 players. This pattern could reflect a potential risk–reward trade-off at the highest competitive level, where even small increases in serve errors might disrupt match outcomes. For elite players, maintaining mental composure and minimizing avoidable serve errors likely represent important tactical considerations. Our findings provide clearer practical implications across different contexts. On grass courts, the positive effect of first serve win rate is particularly strong, highlighting the importance of serve dominance in fast surfaces. On clay courts, consistency in first serve in rate shows greater value, as longer rallies amplify the benefit of stable serve execution. Regarding player ranking, lower-ranked players benefit more from strong first serve win rates, while higher-ranked players rely less on serve-based advantages. At the tournament level, mid-tier competitions emphasize serve stability, whereas high-level tournaments demand more balanced strategies combining serve efficiency with rally performance.

In summary, the empirical findings highlight the need for context-specific serve strategies rather than uniform approaches. Players and coaches should dynamically adjust serving tactics based on ranking, tournament level, and court surface to maximize competitive advantage.These findings also resonate with coaching perspectives and biomechanical evidence. From a coaching standpoint, practitioners often emphasize that excessive reliance on direct-point serves may disrupt tactical rhythm and adaptability, particularly on fast surfaces. Biomechanical studies further suggest that fatigue and stroke mechanics interact with serve effectiveness, which may help explain why first serve win rate does not always exhibit a stable positive effect.Finally, it is important to distinguish between statistical significance and practical significance. Although several serving indicators demonstrate statistically significant causal effects, the corresponding effect sizes often imply only small changes in winning probability, typically within a single-digit percentage range. Such magnitudes may limit their tactical relevance, especially in high-level matches where many other contextual factors interact. Therefore, the tactical implications presented here should be interpreted as preliminary and context-dependent, rather than as decisive strategic prescriptions. Nevertheless, several limitations of this study should be acknowledged. While the DML framework mitigates high-dimensional confounding, it cannot fully eliminate bias from unobservable factors such as player fatigue, psychological state, or in-match tactical adjustments. The exclusive reliance on ATP men’s singles data further restricts the generalizability of the findings to women’s matches, doubles, or junior levels, and may overlook gender- or format-specific serving dynamics. Moreover, although robustness checks were performed, the model specification is still sensitive to unobserved heterogeneity and contextual factors (e.g., critical points, momentum shifts). These limitations suggest that the estimated causal effects should be interpreted as indicative rather than definitive. Future research could extend the dataset to broader competitive contexts, integrate multimodal information (such as biomechanical tracking or psychological measures), and explore complementary causal inference methods to enhance the validity and applicability of the conclusions.

### Future work

Building on the present findings, several directions can advance both the methodological depth and practical relevance of causal analysis in tennis analytics.

1. Methodological Advancement. Future studies could extend the current Double Machine Learning (DML) framework toward dynamic and structural causal models, enabling the analysis of sequential dependencies, momentum shifts, and feedback mechanisms within matches. Incorporating causal mediation or temporal DML approaches would help disentangle indirect pathways—such as how serve performance affects subsequent rally behavior or psychological responses. These developments would refine causal identification and expand the interpretability of estimated effects.
2. Data Expansion and Contextual Integration. Expanding the dataset beyond ATP men’s singles to include WTA, doubles, and junior competitions would enhance the generalizability of causal estimates and allow exploration of gender- and format-specific serving dynamics. Additionally, integrating contextual and match-level variables—such as pressure points, tie-breaks, or decisive sets—would capture how serve effectiveness interacts with psychological and situational stressors, providing a richer understanding of tactical adaptation under varying conditions.
3. Multimodal and Biomechanical Integration. Future research should incorporate multimodal data sources, including biomechanics, player-tracking, and psychological assessments. Combining these modalities would allow for a more holistic model of serving strategies, linking neuro-muscular control, kinematic precision, and tactical decision-making to causal performance outcomes.
4. Predictive and Simulation Applications. Beyond explanatory analysis, causal estimates can be embedded in predictive and simulation-based frameworks. By quantifying the marginal contributions of serve-related indicators, these findings could enhance match-outcome forecasting and inform “what-if” tactical simulations—for instance, testing the effects of increasing serve aggressiveness or improving first-serve consistency under controlled virtual settings. Integrating causal inference with predictive modeling bridges explanation and optimization, offering coaches and analysts an evidence-based foundation for data-driven tactical experimentation.

Collectively, these future directions will deepen the theoretical rigor and practical applicability of tennis analytics, transforming causal inference from a retrospective analytical tool into a forward-looking, decision-oriented framework for performance optimization.

## Data Availability

The datasets used and analyzed during the current study are publicly available from the Tennis Abstract repository (https://github.com/JeffSackmann/tennis_atp).
